# Extubation during extracorporeal membrane oxygenation in severe acute respiratory distress syndrome: time for a paradigm shift?

**DOI:** 10.1186/s13613-023-01214-w

**Published:** 2023-11-29

**Authors:** Alessandro Belletti, Jacopo D’Andria Ursoleo, Anna Mara Scandroglio, Giovanni Landoni, Alberto Zangrillo

**Affiliations:** 1 Department of Anesthesia and Intensive Care, IRCCS San Raffaele Scientific Institute, Via Olgettina 60, 20132 Milan, Italy; 2 School of Medicine, Vita-Salute San Raffaele University, Milan, Italy

We read with great interest the article by Roncon-Albuquerque Jr et al. recently published in *Annals of Intensive Care* [1].

In their prospective study, the authors evaluated safety and feasibility of a standardized approach for extubation during extracorporeal membrane oxygenation (ECMO) in a cohort of 254 patients with severe acute respiratory distress syndrome (ARDS) [1].

In recent years, ECMO has become progressively more popular as a rescue therapy for treating ARDS when refractory hypoxia or hypercapnia is observed. Likewise, the pioneering concept of “awake ECMO” in spontaneously breathing, non-intubated patients is becoming an attractive option for its potential to reduce the incidence of ventilator-induced lung injury (VILI), ventilator-associated pneumonia (VAP) and the side effects of sedative drugs and neuromuscular blocking agents [1, 2].

The authors postulated that the application of a standardized protocol encompassing both clinical and gas exchange criteria as per the Extracorporeal Life Support Organization (ELSO) [3] evaluated on a daily basis combined with careful validation by an intensive care medicine specialist allowed for uneventful extubation during ECMO in 21% of patients with no increased incidence of major adverse events (i.e., refractory hypoxemia, cardiac arrest, and accidental decannulation).

We commend the authors for conducting this study, which reports on the largest experience available so far in the literature dissecting the feasibility of “awake ECMO” to treat non-coronavirus disease 2019 (COVID-19)-related ARDS refractory to invasive mechanical ventilation (IMV). We were pleased to note that the authors reported a failure of extubation during ECMO in only 11 patients (20%) within the ‘EXT group’.

Our group recently completed a comprehensive systematic review encompassing 57 studies (467 “awake ECMO” patients) which reported the outcomes of “awake ECMO” in patients with respiratory failure [2].

Within the ARDS subgroup (62 patients), the pooled estimate for “awake ECMO” failure was 39.3% (95% CI 24.0–54.7%), higher than reported by the authors.

We hypothesize that careful selection of patients and application of a strict protocol allowed the authors to halve the extubation failure rate as compared with previous literature. Main reasons for awake ECMO failure, as reported in pertinent articles, were worsening respiratory failure, delirium/agitation, bleeding from the airways and lack of secretion clearance, which is consistent with the authors’ findings [2].

Unfortunately, to date only few studies report detailed criteria for awakening and weaning from the IMV while still on ECMO support [4].

In this regard, to the best of our knowledge, the study conducted by the authors was the first to investigate the ELSO criteria for extubation during ECMO [3], and the relatively low rate of extubation failure observed may be indirect evidence to support validity of the ELSO criteria to select candidates for “awake ECMO”.

We have the following comments as well.

Based on the protocol, also extubated patients remained mildly sedated during ECMO course.

Furthermore, it is unclear whether patients underwent active physiotherapy while on ECMO. We believe that active physiotherapy may provide additional advantages and potentially elicit a key difference between the “conventional” and “awake ECMO” management.

We also noticed that several key baseline parameters were not provided. Specifically, additional information on parameters of right heart function and multi-organ failure (e.g., renal-replacement therapy, liver failure, etc.) would be of great help to further characterize key baseline differences between the two groups and hence to better identify selection criteria.

Lastly, though it seems that gas exchange criteria were mainly used to select patients for extubation, we believe that additional right ventricular function and respiratory mechanics parameters should be considered as well when selecting patients, and also further investigated as potential success predictors.

Consequently, while substantial preliminary evidence reinforces the idea that “awake ECMO” is undeniably feasible in selected patients with a low failure risk even in the ARDS setting, we acknowledge that finding the most accurate criteria for an optimal management approach to extubate patients who still require ECMO support remains to be determined.

Our group recently provided evidence that individuals with COVID-19-induced ARDS, showcasing the Macklin effect on chest computed tomography (CT) scans (characterized by linear air accumulation along bronchovascular bundles, visceral pleura, and/or interlobular septa), were at a higher risk of developing pneumomediastinum and/or pneumothorax [5].

In such cases, tailoring the respiratory support according to the patient respiratory effort intensity could be a possible approach for mitigating the progression of the Macklin effect. In particular, individuals in this group may find benefit from the use of ECMO without IMV [5].

Therefore, early detection of the Macklin effect (identified on chest CT scans) could be incorporated into patient selection criteria to assist the intensive care medicine specialist in choosing the ideal candidate for “awake ECMO”.

Nevertheless, additional prospective studies and a broader range of experiences are needed to validate the efficacy and safety of the “awake ECMO” approach for ARDS patients. This will enable better selection and stratification of individuals for whom the spontaneous breathing ECMO strategy could be beneficial and secure.

## Data Availability

Not applicable. This letter does not report original data.
